# Are MSCs angiogenic cells? New insights on human nestin-positive bone marrow-derived multipotent cells

**DOI:** 10.3389/fcell.2014.00020

**Published:** 2014-05-20

**Authors:** Simone Pacini, Iacopo Petrini

**Affiliations:** Department of Clinical and Experimental Medicine, University of PisaPisa, Italy

**Keywords:** mesenchymal stromal cells, endothelial differentiation, angiogenesis, adult stem cells, bone marrow, nestin, neo-vascolarization, *in vivo* MSC

## Abstract

Recent investigations have made considerable progress in the understanding of tissue regeneration driven by mesenchymal stromal cells (MSCs). Data indicate the anatomical location of MSC as residing in the “perivascular” space of blood vessels dispersed across the whole body. This histological localization suggests that MSCs contribute to the formation of new blood vessels *in vivo*. Indeed, MSCs can release angiogenic factors and protease to facilitate blood vessel formation and *in vitro* are able to promote/support angiogenesis. However, the direct differentiation of MCSs into endothelial cells is still matter of debate. Most of the conflicting data might arise from the presence of multiple subtypes of cells with heterogeneous morpho functional features within the MSC cultures. According to this scenario, we hypothesize that the presence of the recently described Mesodermal Progenitor Cells (MPCs) within the MSCs cultures is responsible for their variable angiogenic potential. Indeed, MPCs are Nestin-positive CD31-positive cells exhibiting angiogenic potential that differentiate in MSC upon proper stimuli. The ISCT criteria do not account for the presence of MPC within MSC culture generating confusion in the interpretation of MSC angiogenic potential. In conclusion, the discovery of MPC gives new insight in defining MSC ancestors in human bone marrow, and indicates the *tunica intima* as a further, and previously overlooked, possible additional source of MSC.

## Discover, isolation, and characterization of MSCs

In the late sixties, A. J. Friedenstein and coworkers first described the multipotent mesenchymal stromal cells (MSCs). When human bone marrow (BM) cells were cultured in plastic dishes colonies of adhered fribroblastoid cells proliferate and hematopoietic precursors progressively disappear. These cells have been named C*olony Forming Units of Fibroblastoid cells* (CFU-Fs) because of their ability to form large colonies on plastic surfaces. MSCs are able to differentiate into chondrocytes and osteoblasts, *in vitro* (Friedenstein et al., 1968), and *in vivo* (Friedenstein et al., 1974). By that time, T. M. Dexter and colleagues developed a culture system to study hematopoiesis and demonstrated that bone marrow hematopoietic stem cells (HSCs) were unable to adhere onto the culture flasks but necessitate an underlying layer of adherent cells that mimics the bone marrow stromal compartment (Dexter et al., 1977). After the demonstration that CFU-Fs originate from the bone marrow stroma, their name was changed in bone marrow stromal cells (Lanotte et al., 1981). In 1991, A. I. Caplan suggested the presence of a stem cell population in the adult BM able to differentiate into multiple mature cell lineages sharing a common precursor in the mesodermal layer of the embryo. Therefore, these cells were named “mesenchymal stem cells” (Caplan, 1991). Subsequently, the differentiation potential of MSCs into multiple mature lineages has been confirmed: these cells have a stable phenotype that can be easily expanded in culture and maintain the ability to differentiate into osteoblasts, chondrocytes, adipocytes, tenocytes, myocytes and stromal cells supporting hematopoiesis (Pittenger et al., 1999). MSCs became popular when K. Le Blanc et al. showed that MSCs are “invisible” to the immune system because they express only the class I Major Histocompatibility Complex (MHC-I) but not the class II and co-stimulatory molecules such as CD40, CD80, and CD86. This is relevant in the prospective of allogeneic transplantation with possible therapeutic applications (Le Blanc et al., 2003).

The isolation and the expansion of MSCs are easily feasible from the adult bone marrow in most cellular laboratories. Therefore, MSCs have been largely evaluated and employed in several premature clinical trials. However, a precise characterization of these cells is still missing as well as standardized protocols for their isolation and expansion. Consequently, results are controversial and the biology of MSCs is still unclear.

More recently, the International Society for Cellular Therapy (ISCT) has proposed minimal criteria to define MSCs (Dominici et al., 2006):

1. MSCs are plastic-adherent cells in standard culture conditions,
2. MSCs express the surface marker: CD105, CD73, and CD90 and do not to express the hematopoietic markers: CD45, CD34, CD14, and CD11b, and
3. MSCs must be able to differentiate toward osteogenic, adipogenic and chondrogenic lineages when exposed to the proper conditions *in vitro*.

Finally, MSCs have been re-named “mesenchymal stromal cells” modifying the term “stem” into “stromal” in order to maintain the same acronym and avoid possible over-interpretation of their limited pluripotent potential (Horwitz et al., 2005).

## Tissue origins and distribution of MSCs

Cells matching the ISCT criteria can be isolated not only from the bone marrow but also from other adult and the fetal tissues (De Bari et al., 2001; Zuk et al., 2001; In't Anker et al., 2004; Seo et al., 2004). In these studies the culture condition adopted to establish BM-derived MSCs were applied to cells isolated from other tissues, in order to determine if MSCs reside also in different organs. In mice, L. da Silva Meirelles et al. demonstrated that long-term culture of MSCs could be established from a wide range of different adult tissues including fat, muscles, pancreas, vena cava, kidney glomerulus, aorta, brain and many others alongside bone marrow (da Silva Meirelles et al., 2006). Notably, all the cell populations that L. da Silva Meirelles established, independently from their origin, were long-term culture of adherent cells, with MSC phenotype and able to differentiate into mesenchymal cell lineages. These data suggest the presence of MSCs in virtually in all organs and tissues of the body. Three hypotheses try to explain the tissue distribution of MSCs:

1. MSCs permanently reside in multiple tissues and organs,
2. MSCs reside in only in particular tissues but can circulate in blood, and
3. MSCs are circulating blood cells.

The presence of CFU-Fs in blood of adult mammals was shown in 2007 (He et al., 2007). However, the contamination with fragments of connective tissue could not be ruled out to justify the presence of MSCs in the collected sample. Disputes remain regarding the existence of circulating MSCs (Roufosse et al., 2004; Kuznetsov et al., 2007). L. da Silva Meirelles demonstrated the presence of MSCs within tissue cleaning the organs using intravascular perfusion before their collection. Nonetheless, the possibility that MSCs may circulate locally or systemically under non-physiological conditions, i.e., tissue injury, is not excluded. Although, the features of MSC from different organs are similar, mild differences in differentiation potential and surface markers have been reported. These differences have been related to the influence of a modified local environment (niche) present in different site of the body.

MSC can be isolated from the wall of blood vessels (Doherty et al., 1998; Bianco et al., 2001), and in 2007, B. Sacchetti et al. demonstrated a common phenotype for BM-derived CFU-Fs and Adventitial Reticular cells (ARCs). ARCs populate sinusoids and lay in close contact with the endothelium. Strong evidence indicate that the fibroblastoid colonies described by Friedenstein *in vitro* originate from the ARCs isolated *ex vivo* (Sacchetti et al., 2007). Stromal progenitors in human BM that reside in the sub-endothelial layer of sinusoids strongly express the melanoma-associated adhesion molecule (MCAM/CD146). A. Tormin et al. confirmed that CD271 is an *in vivo* marker of BM-derived MSCs (Quirici et al., 2002) and described a subset of CD271+ cells that express CD146. Cells expressing CD271 and CD146 are the ARCs present in the sub-endothelial layer of sinusoids. The remaining CD271+/CD146− cells maintain the MSC features but *in vivo* reside in the trabecular bone-lining endosteal niche (Tormin et al., 2011). Therefore, there are at least two cells able to generate MSCs from the bone marrow one in the perivascular (CD271+/CD146+) and one in the endosteal niche (CD271+/CD146−).

M. Corselli et al. isolated two distinct MSC progenitors from the stroma vascular fraction (SVF) of the adipose tissues (Corselli et al., 2012). CD34−CD146+ pericytes encircling capillaries and microvessels and CD34+CD146− adventitial cells surrounding larger arteries and veins. MSC-like cultures can be expanded from both these populations, suggesting a vascular origin for the MSCs of the adipose tissue, similarly to what observed in bone marrow. A hierarchical organization of cell differentiation has been proposed for the vascular progenitor of MSCs being the adventitial cells the precursors of pericytes. Indeed, under proper conditions, ARCs differentiate into pericytes *in vitro*. Later, M. Crisan et al., isolating the cells through CD146, demonstrated the perivascular origin of MSCs in multiple organs (Crisan et al., 2008).

A new hypothesis that MSCs are localized *in vivo* in “perivascular” spaces that extend through the whole post-natal organism has been proposed. While this latest hypothesis is gaining consensus among researchers, the term “perivascular” is somehow ambiguous because it include the proximity of vessel and the wall itself. More precisely, two intra-vessel wall compartments, the *adventitia* and *sub-endothelium*, have been indicated as possible locations for these two MSC progenitors. The relationship between these two progenitors remains obscure, even if they have been largely characterized. Their histological localization, suggests a role of MSC in blood vessel formation *in vivo*. MSCs can directly differentiate into vascular cells (endothelial cells and smooth muscle cells) and/or as supporting vascular (re)-generation in response to the paracrine secretion of stimulating factors (Lin and Lue, 2013).

## Controversies about the angiogenic potential of MSCs

One of the most interesting debates regarding MSCs concerns their angiogenic potential. Due to the possible role of MSC in therapeutic (re)-vascularization, an increasing number of studies *in vitro* and *in vivo* have been performed (reviewed in Vittorio et al., 2013).

The formation of new blood vessel can be divided in:

1. Vasculogenesis: *de novo* formation of blood vessels from the endothelial precursors or angioblasts,
2. Angiogenesis: includes sprouting of existing vessels and intussusceptive angiogenesis, and
3. Arteriogenesis: remodels a pre-existing collateral circulation (Makanya et al., 2009; van Royen et al., 2009; Melero-Martin et al., 2010; Carmeliet and Jain, 2011; Potente et al., 2011).

Although MSCs support these processes through the release of angiogenic factors and protease (reviewed in Watt et al., 2013), the relevance of their differentiation into endothelial lineages is still debated.

The demonstration of MSC commitment toward endothelial lineage is often limited to the detection of the upregulation of typical EC surface molecules including CD31, CD34, VEGF receptors (VEGFR1, VEGFR2) and von Willebrand factor (vWF). As phenotype modification is insufficient to demonstrate differentiation, additional functional tests are often performed. These tests include *in vitro* tube formation on Matrigel® and uptake of acetylated-low density lipoproteins (Ac-LDL). Nonetheless, it might be inaccurate describing these differentiated MSCs as fully mature and functional ECs basing on these *in vitro* assays. For example, Ac-LDL uptake has been described also in macrophages and pericytes (Voyta et al., 1984).

A large effort was spent for the optimization of protocols able to induce endothelial differentiation of MSCs. VEGF stimulates the differentiation of MSCs into ECs. In 2004, J. Oswald et al. demonstrated that confluent human BM-derived MSCs cultured in 2% fetal calf serum (FCS) and 50 ng/ml VEGF for a week, displayed upregulation of endothelial surface markers including VEGFR1, VEGFR2, VE-Cadherin, VCAM-1, and vWF. Moreover, when incubated on Matrigel® *in vitro*, MSCs formed characteristic capillary-like structures (CLS) (Oswald et al., 2004). Similarly, M. Jazayeri et al. cultured human BM-derived MSCs in medium supplemented with 5% FCS, IGF and VEGF and detected CD31, vWF, Tie2, VCAM1, and VE-cadherin on the cell surface. In addiction, applying electron microscopy Authors showed the presence of typical EC morphological features including Weibel-Palade bodies, tight junctions and caveolae (Jazayeri et al., 2008). Similar results were also achieved using “endothelial growth medium-2” (EGM-2, which contains VEGF, EGF, FGF-2, IGF-1, hydrocortisone, heparin, ascorbic acid and 2% FCS) (Liu et al., 2007) and MSCs isolated from the adipose tissue (Cao et al., 2005; Fischer et al., 2009). Conversely, V.D. Roobrouck et al. reported that VEGF treatment of human BM-derived MSCs significantly increased mRNA expression of CD34, VEGFR1, and VEGFR2, but not of Tie-2 and vWF or CD31 that was even decreased. These MSCs also failed forming CLS in Matrigel® assays, (Roobrouck et al., 2011). In parallel, W. Fan et al. demonstrated that human BM-derived MSCs cultured in the presence of different concentrations of VEGF did not show increase in CD31, vWF or VEGFR2 expression (Fan et al., 2011).

*In vivo* G. V. Silva et al. demonstrated that MSCs applied in a region of myocardial ischemia can differentiate into smooth muscle cells and endothelial cells leading to increased vessel density and an improvement the cardiac function, in a canine model (Silva et al., 2005). Nonetheless, there is a consolidating concept that the angiogenic effect of MSCs is predominantly caused by their paracrine actions rather than their EC trans-differentiation potential. A. Al-Khaldi et al. showed that, in the murine Matrigel® plug assay, more than 99% of the new-formed blood vessels originated from host-derived EC, while a small portion of injected BM-derived MSCs were found in the close proximity of- or within blood vessels (Al-Khaldi et al., 2003). Moreover, the observation that MSCs *in vitro* committed through endothelial lineages were not superior to “*naïve*” MSCs in stimulating *in vivo* angiogenesis, may underline the relevance of the secretion of pro-angiogenic factors that is also sustained by uncommitted MSCs (Liu et al., 2007; Fan et al., 2011). Thus, the up-regulation of endothelial marker, under specific culture conditions, could represent an *in vitro* artifact and not a real differentiation into functional endothelial cells. BM-derived MSCs seems to be an important regulator of neo-vascularization by the secretion of pro-angiogenic factors as well as by differentiating into functional pericytes able to stabilize the new-formed vasculature (Au et al., 2008), rather than a source of endothelial progenitor cell.

Most of the controversial data about endothelial differentiation of MSCs need to be discussed as consequence of the sub-optimal protocols of differentiation (Janeczek Portalska et al., 2012). Moreover, a critical issue is the heterogeneity of the primary MSC cultures used to generate endothelial progenitors.

## Heterogeneity of culture expanded MSCs

The anatomic localization and the physiological function of MSCs are not clearly characterized. MSCs are commonly isolated from long-term cultures; therefore, it remains difficult to determine the primary cells of origin. The loose ISCT criteria hamper the identification of unique precursors of MSCs. Indeed, several types of primary cells with different features can fulfill the definition of MSCs *in vitro*. Being the definition permissive, the presence of a unique common precursor for cells with MSC features cannot be hypothesized. In BM, MSCs can originate from both perivascular and endosteal progenitors, therefore, it is difficult to distinguish if there is a unique common precursor or if the loose ISCT definition is unable to identifies two different progenitor populations. However the clinical applications of MSCs are only partially limited by the incomplete characterization of the progenitor cells.

The heterogeneity of MSC cultures, defined according to ISCT criteria, is becoming evident in more recent articles and brings into question the utility of these ambiguous criteria. From the beginning, different terms have described the morphology of plastic-adherent cells: fibroblastoid (Werts et al., 1980), giant fat cells and blanket cells (Allen and Dexter, 1983), spindle shaped flattened cells (Kuznetsov et al., 1997) and very small round cells (Colter et al., 2001). Thus, mesenchymal cell morphology seems to be highly dependent on culture conditions: supplements, seeding density, number of passages and culture time (Wagner and Ho, 2007; Barachini et al., 2009). It is still unclear if there is any relation between these different morphology and cell functions.

There is not a consensus on the surface markers of MSCs, aside from the unspecific CD105, CD90, CD44, and CD73, because different laboratories use different sets of antigens. Therefore, the differentiation into mature cells with a mesengenic ancestor seems to be the more reliable and stringent criteria to define MSCs. However, the differentiation potential of MSC is variable. This variability is observed between different donors (Phinney et al., 1999) and also within different colonies obtained from the same subject (Russell et al., 2010). Indeed, colonies obtained from the same individual could be characterized as mono-, bi- or tri-potent on the basis of their ability to differentiate into, respectively one, two or three types of tissue: osteogenic, chondrogenic and adipogenic lineages. Moreover, it has been clearly demonstrated that repeated passages progressively reduce the multi-lineage differentiation ability, introducing a further element of complexity (Muraglia et al., 2000). It is possible to hypothesize that the angiogenic potential of MSC is subject to a similar variability that is influenced by the same factors.

MSCs are heterogeneous not only among different colonies but also within the same colony (Digirolamo et al., 1999). Cells show variable differentiation potential in relation to their topographic localization inside the colony. Cells from the center and the margins of the colony differ for shape, differentiation potential and surface markers (Ylöstalo et al., 2008; Sengers et al., 2010). Therefore, the term “multipotent mesenchymal stromal cells” does not identify a population of cells with uniform features and unambiguous potential but refers to a highly heterogeneous population that is dramatically affected by donors characteristics (Russell et al., 2013), isolation methods (Wagner and Ho, 2007; Barachini et al., 2009), culture conditions (Bieback et al., 2009).

Several possible mechanisms may explain the basis of the MSC heterogeneity, beside the already described variability introduced by *ex vivo* procedures (Pevsner-Fischer et al., 2011). Hypotheses on the origin of this variability include: stochastic events, occurring during expansion and differentiation and a possible *in vivo* heterogeneity of the isolated cell populations. In this latter hypothesis, specific culture conditions select, or simply promote, particular subpopulations of MSCs giving reason of the observed heterogeneity and the morpho-functional variability. According to this scenario, numerous multipotent cell populations can be described in bone marrow, some of them able to differentiate into lineage from the three germ layers: endoderm, mesoderm and ectoderm (triploblastic differentiation). However, the isolation and successive characterization of these cells is strictly dependent on the application of specific culture conditions. For example, “marrow-isolated adult multipotent inducible (MIAMI)” cells can differentiate into, neural and pancreatic-like cells in addition to skeletal tissue lineage (D'Ippolito et al., 2004). The isolation and expansion of MIAMI cells require specific culture conditions with low oxygen tension.

Recently a unique multipotent sub-population in adult human BM-derived MSCs has been isolated using fluorescent activated cell sorting (FACS) for stage-specific embryonic antigen 3 (SSEA-3). BM-derived MSCs show poluripotency-differentiation properties (Kuroda et al., 2013). Interestingly, stress conditions could enrich the expression of SSEA-3 in cultured MSCs (Kuroda et al., 2010). Y. Kuroda et al. demonstrated that long-term trypsin incubation could increase the recovery of cell clusters containing pluripotency-associated markers and renamed these cells as “multi-lineage differentiating stress-enduring” (MUSE) cells.

Thus, if mild modifications in the culture conditions, or in the culture procedures, can induce/preserve an embryonic-like differentiation potential in BM-derived cells, it is reasonable to suppose that angiogenic potential behaves similarly and is significantly affected by manipulation *in vitro*.

## Mesodermal progenitor cells (MPCs) in adult human bone marrow

In 2007, we attempted to optimize MSC culture conditions for clinical application selecting media without supplements of animal origin. When medium was supplemented with autologous serum instead of that of bovine origin, a small population of cells with distinct shape was noticed. These cells presented rounded, fried-egg shape, instead of the usual spindle morphology of MSCs, were highly refractive and remain firmly attached to the plastic during trypsin digestion

In 2008, by replacing fetal bovine serum (FBS) with pooled human AB serum (PhABS) in the culture medium of human BM cells, we were able to characterize this new population of adherent cells (Petrini et al., 2009). These cells are quiescent: Ki-67 negative; with long telomeres and express the pluripotency-associated transcription factors Oct-4 and Nanog instead of RUNX2 and Sox9 typical for MSC-phenotype (Pacini et al., 2010). Phenotypically, these cells share the expression of CD105 with MSCs but lacked expression of CD73, CD90, CD166, CD271 and those other markers typical of the mesenchymal phenotype such as MSCA-1. Interestingly, this cell population rapidly produces mesenchymal offspring when supplemented with FBS or human cord blood serum. Thus, this novel population of cells, isolated from the BM, has *in vitro* characteristics of a progenitor of the mesengenic lineage and therefore has been named “*Mesodermal Progenitor Cells*” (MPCs).

### Isolation of MPCs from human BM samples

Method for the isolation of MPCs from BM samples is feasible, inexpensive and based on selective culture conditions (Trombi et al., 2009). Initially, MPCs were co-isolated together with MSCs applying media supplemented with autologous serum or PhABS, in culture. It became evident that MPCs have different adhesion properties compared to MSCs. In fact, applying standard trypsin-based cell detaching protocols, MSCs were entirely harvested while most of the MPCs remain firmly attached to the plastic surface and required different proteases' solution (TrypLE Select® from LifeTechnologies) to be detached. Therefore, plastic features and coating of the culture surfaces influence the proportion of MPCs and MSCs in the primary cultures. We firstly tested not gas-treated hydrophobic plastics, usually applied for cultures in suspension, and surprisingly MPCs were able to attached on that surface also. Conversely, the hydrophobic conditions resulted not permissive for MSC. Thus, a selective culture could be established using these conditions allowing the recovery of MPCs with a purity of more than 95%. We also noticed that a higher yield of MPC recovery was achieved using higher seeding densities, than that usually applied for MSC isolation. In summary, PhABS supplementation and high density seeding on hydrophobic plastics were the selective culture conditions necessary for MPCs isolation from BM-MNCs. This method has been consolidated and it is highly reproducible allowing the quality screening of the MPC preparations before their employment in the different studies.

The mechanisms behind the difference, in MPC recovery, between culturing cells in FBS or PhABS-containing medium are still unknown. Nonetheless, we demonstrated that the addition of PhABS, even in small percentages, to FBS primary cultures allows MPC isolation, suggesting the presence of undetected agents able to induce MPCs in the human serum (Trombi et al., 2009). Conversely, when FBS is added to cultures grown in PhABS, cells differentiate into MSCs without any significant reduction in the number of MPCs indicating a possible semi-conservative proliferation of MPCs. What characterized FBS against PhABS is the different origin in terms of species (bovine instead of human) and stage of body development (fetal against adult). The differentiation of MPCs into MSCs is induced replacing PhABS with FBS (Petrini et al., 2009), or using human cord blood-derived serum (unpublished data). These preliminary results suggest that media supplementation with fetal sera represent the culture condition for the mesengenic induction of MPCs, independently from the adopted species.

#### Mesengenic potential of MPCs

**Figure 1:**
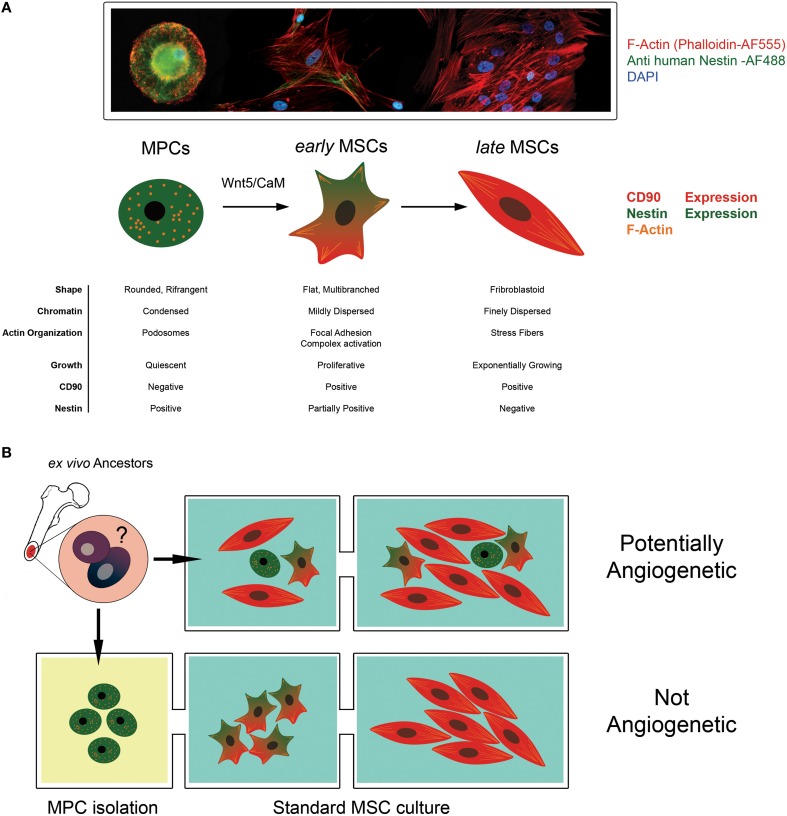
**The angiogenic potential of MSC cultures is controversial. (A)** Mesodermal Progenitor Cells (MPCs) are CD90-, Nestin+ progenitors of MSCs. These cells remain in a quiescent state presenting a typical fried egg-shape, condensed chromatin and podosomal structures. When mesengenic differentiation is induced, MPCs differentiate into “*early* MSCs.” *Early* MSCs slowly proliferate, express Nestin and CD90 (a MSC marker) and modify their shape because of Actin re-organization in focal adhesion complexes. Under persistent stimulation toward mesengenic differentiation, “*early* MSC” become “*late* MSC” showing exponentially growth, fibroblastoid shape and the ability to differentiate into skeletal tissues (fat, bone and cartilage). **(B)** According to this hierarchical model, MPCs can be considered a putative progenitor of the mesenchymal lineage *in vivo*, being present in the bone marrow mononuclear fraction. When supplemented with media containing FBS, these cells rapidly differentiate toward the mesengenic lineage and form asynchronous and heterogeneous cultures fulfilling the ISCT MSC criteria of definition. The undetected and unpredictable presence of sub-populations of MPCs, in culture could explain the variable angiogenic potential described for MSC in the literature. Conversely, the isolation of MPC and their subsequent differentiation using optimized protocol can allow the generation of synchronized and homogeneous mesenchymal stromal cells with a reproducible angiogenic potential.

Later, we have been able to demonstrate a hierarchical multi-step model of mesenchymal differentiation with at least three different populations of multi-potent cells (Fazzi et al., 2011). Indeed, MPCs can generate exponentially growing MSC cultures after 2 weeks of stimulation with differentiating conditions. The differentiation proceeds through the commitment into an intermediate cell population; we named *early* MSCs. Timing of MPC mesengenic differentiation was definitively clarified and specific morphologies, phenotypes and growing features of the three protagonists described (Figure 1A). Studying Wnt signaling activation during MPC differentiation, we showed that non-canonical Wnt5/Calmodulin pathway was involved in the commitment of MPCs into *early* MSCs and demonstrated that Calmidazolium Chloride, a Calmodulin inhibitor, was able to interfere with the differentiation only at this initial step while has no effect on passage from *early* to *late* MSCs.

#### Angiogenic potential of bone marrow-derived MPCs

From the beginning it has been clear that MPCs have angiogenic potential because they form capillary-like structures (CLS) after a multiple steps of differentiation (Petrini et al., 2009; Trombi et al., 2009; Pacini et al., 2010). Interestingly, the inhibition of the Wnt5/Calmodulin signaling pathway has no effects on the MPC differentiation toward endothelial lineage suggesting that Wnt signaling pathway activation finely regulates MPC fate. On the contrary of mesengenic, all the passage of angiogienic differentiation have not been described to date due to the lack of specific culture protocols. Indeed, to partially induce endothelial differentiation we applied protocols optimized for endothelial progenitor cells (EPCs) with mild modifications (Hill et al., 2003). This was sufficient to demonstrate the angiogenic potential of MPCs and suggested that exist a BM-derived endothelial progenitor cell distinct from EPCs.

The high expression of CD31 and Nestin in MPCs suggest the existence of a primitive progenitor for the endothelial lineages (Petrini et al., 2009; Trombi et al., 2009; Pacini et al., 2010). Nestin is a class VI intermediate filament protein originally described as a marker of neural stem cells that is expressed during the development of central nervous system (CNS) (Lendahl et al., 1990). Although Nestin expression is down regulated during the differentiation into neurons or glial cells (Dahlstrand et al., 1995), it can be detected in adult neural progenitor cells (Reynolds et al., 1992; Morshead et al., 1994) and in some CNS tumors (Tohyama et al., 1992). Frequently, Nestin is not expressed by the cancer cells themselves but can be observed in the endothelial cells of the tumor regardless of malignancy grade or its histotype (Sugawara et al., 2002). This suggests that Nestin can be a marker of proliferating tumor endothelial cells and not only of neuroepithelial elements. Therefore, Nestin expression correlates with angiogenesis because it is expressed in proliferating vascular endothelial cells of the tumor (Kim et al., 2002; Teranishi et al., 2007; Gravdal et al., 2009; Eaton et al., 2010). K. Sugawara et al. demonstrated a high expression of Nestin in bovine aortic proliferating endothelial cells in static culture. This expression rapidly decreases under conditions of laminar shear stress flow, suggesting that Nestin expression is typical of early proliferating endothelial precursors but is loss in mature endothelial cells of normal tissues. In the recent years, the emerging concept of Nestin as a novel early angiogenic marker is gaining consensus in normal ad tumor angiogenesis (reviewed in Matsuda, 2013).

MPCs express CD31/PECAM (Pacini et al., 2013) but rapidly loose these markers during differentiation contextually with the lost of Nestin (Figure 1A). Preliminary data show that MPCs can make “sprouting” when directly seeded in Matrigel® 3D-cultures but are not able to efficiently form CLS without a step of pre-differentiation. Therefore, MPCs could represent a very staminal progenitor with angiogenic potential more immature than what is reputed to date.

## MPCs do not show features of pericytes or adventitial progenitors

Pericytes surround blood capillaries, precapillary arterioles, post-capillary venules and collecting venules (Sims, 1986; Allt and Lawrenson, 2001); where they can be identify by the expression of CD146 together with less specific markers such as α-smooth muscle actin (α-SMA), desmin, NG-2, platelet-derived growth factor receptor (PDGFR)-α, aminopeptidase A and N, RGS5, and the promotertrap transgene *XlacZ4* (Gerhardt and Betsholtz, 2003).

Pericytes and Adventitial Progenitor Cells (APCs) belong to the same cell lineage, according to several authors (Tormin et al., 2011; Corselli et al., 2012). Pericytes and APCs differ in their position in the vessel wall morphology, and some surface markers. However, this distinction is not absolute because exist a continuum between the phenotype of the classical APCs and the typical pericytes when they are distributed along small vessels such as arteriole, capillary, and venule. It has been suggested that pericytes may reside under the endothelium even in large vessels and support the endothelial removal and repair after injuries. From this point of view, these cells can be considered a reservoir of MSC-like undifferentiated cells (da Silva Meirelles et al., 2006). Conversely, Corselli's data demonstrated that APCs differentiate into pericytes *in vitro*, suggesting a “centripetal” (from the *adventitia* toward sub-endothelial layer) relationship between these cells (Corselli et al., 2012). Both the “centrifugal” and the “centripetal” model assumed that the *intima* would not be involved in the generation of MSC-like cells.

**Figure 2:**
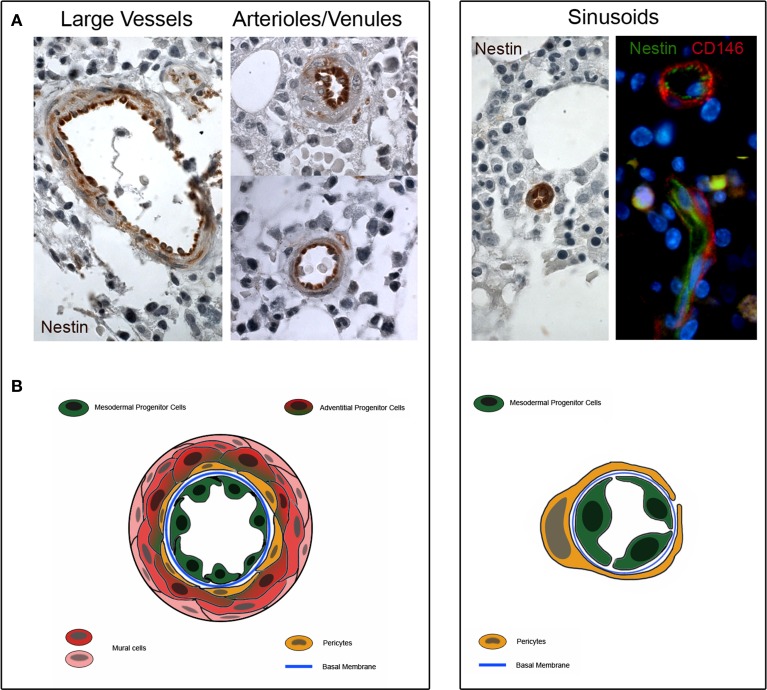
**Nestin expression in human bone marrow biopsies. (A)** Consistent expression of Nestin is detectable in the *tunica intima* of large vessels, arterioles and venules (dark brown color). Few cells of the sub-endothelium and *adventitia* show a weakly positive Nestin staining (light brownish color). Furthermore, Nestin is highly expressed in the sinusoids and two colors immunofluorescence reveals that Nestin is positive in lumen-facing cells surrounded by CD146+/Nestin− pericytes. **(B)** Schematic representation of possible tissue distribution of MPC (represented in green) and other bone marrow vessels-residing cells.

Interestingly, our MPCs express the early angiogenic markers Nestin and CD31/PECAM, suggesting their plausible location in the *tunica intima*. Although the localization of MPCs in the lumen-facing wall of vessels has been not definitively demonstrated, preliminary histological evaluations revealed the expression of Nestin in the sinusoids, arterioles/venules and larger vessels of bone marrow (Figure 2). A higher Nestin expression was observed in sinusoids' endothelium, whereas a lower Nestin expression was localized in only few cells located in the sub-endothelium and *adventitia*.

MPC do not express CD146, a specific markers of pericytes, as well as CD271. Therefore, it is unlikely MPCs reside in the *adventitia* or in the sub-endothelium (Petrini et al., 2009; Trombi et al., 2009; Pacini et al., 2010). Thus, any model of perivascular localization of MSC-like cells will be incomplete without the inclusion of the *tunica intima* among the possible sources of these cells.

## New hypothesis on angiogenic properties of BM-derived multipotent stromal cells

Recently, we have proposed that the yield of MPCs, co-isolated in the standard BM-MSC preparations, is influenced from the host, the batches of the serum and from the density of cell seeding (Petrini et al., 2009). The inter-population variability introduced by different donors and cell isolation protocols, affect the yield of MPCs, *early* MSCs or *late* MSCs. According to the hierarchical model, MPCs are progenitors of the mesenchymal lineage and account for 1–3% of mononuclear cells of the bone marrow (BM-MNCs). When BM-MNCs are seeded in standard FBS-containing media MPCs rapidly differentiate into mesengenic lineages forming the typical MSC culture in few days.

The variable angiogenic potential described in the literature of MSCs is probably related to the heterogeneous composition of the cultures expanded from the bone marrow that includes sub-populations of MPCs and MSCs when defined according to ISCT criteria (Figure 1B). Indeed, the expanded or exponentially growing MSCs are Nestin-negative and *de facto* coincide with our *late* MSCs. Late MSCs do not retain any angiogenic potential because these cells are already committed toward other mesengenic lineage (Figure 1B). Most of the reports do not apply specific protocols for MPC's isolation; thus, these MSC cultures represent an uncontrolled heterogeneity of multipotent cells with an unpredictable angiogenic potential. Because MPCs show resistance to trypsin digestion, these cells are expected to be lost during subsequent passages reducing progressively the angiogenic potential of the sub-cultures. Not surprisingly, the most successful endothelial differentiation protocols have been obtained from early passages of MSC's cultures (Oswald et al., 2004; Fan et al., 2011; Janeczek Portalska et al., 2012). Conversely, protocols specific for endothelial differentiation may commit a pure population of MPCs into homogeneous clones of MSCs. The clinical utility of BM-derived cells will be improved by a more precise phenotypization able to distinguish MPCs from *early* and *late* MSCs.

Recently, Frenette's group demonstrated in the hematopoietic niche the presence of Nestin-positive bone marrow cells with mesengenic potential of differentiation. Using *Nes-Gfp* transgenic mice, authors identified a highly selected fraction of MSCs able to form the HSC niche (Méndez-Ferrer et al., 2010). *In vivo*, both in human and mouse, these cells are positive for PDGFRα, CD51 and Nestin expression and negative for CD45 CD31 CD235a (Ter119- in mice). Although the two types of BM stem cell can form a single niche, only a small fraction of Nestin+ cells exhibits MSC activity when tested in mesensphere or CFU-F assays (Méndez-Ferrer et al., 2010). Also for Nestin-positive cells, the protocols of isolation and expansion dramatically affect the composition of cell populations in culture; thus harsh isolation protocols will be needed to avoid the development of heterogeneous populations and allow the characterization and functional definition of these interesting cells. Further the co-expression of PDGFRα and CD51 identifies a subset (about 60%) of Nestin-positive cells with an enriched potential to form HSC niches and to perform mesenchymal differentiation (Pinho et al., 2013). Limited data are available regarding the angiogenic potential of Nestin-positive cells. Because PDGFRα+ CD51+ hematopoietic-supporting stromal cells do not express CD45 (a hematopoietic marker) and CD31 (an endothelial marker), it can be hypothesized the presence of an additional population of Nestin-positive CD31-positive cells not described in the previous analysis.

## Conclusions

Although MSCs have been largely studied for their interesting applications in clinical trials, these cells have not been fully characterized because of the lack of standardized protocols between different laboratories. Controversies remain, and several aspects of MSC biology are still unclear. The heterogeneity and morpho-functional variability of MSC cell preparations could explain most of the conflicting data in the literature. Together with the effects of culture conditions that can indeed select, or simply promote, particular subpopulations of MSC-like cells (Table 1), the described possible multiple origins of MSCs contribute to the confusing interpretation of the experimental data. More stringent phenotypization criteria may help to prevent this issue. Recently, perivascular localization of MSC precursors may explain their presence in a wide range of tissues and organs and suggests some angiogenic potential.

**Table 1:** ***Bona fide* multipotent progenitor cells of bone marrow and adipose tissues**.

**Acronym**		**MSCs**	**MPCs**	**Pericytes**	**ARCs**	**APCs**	**TBLCs**
Phenotype							
	CD90	+	−	+	+	+	+
	CD105	+	+	+	+	+	+
	CD73	+	−				
	Nestin	±	+		±		
	CD31	−	+	−	−	−	−
	CD146	±	−	+	−	−	±
	CD271	+	−	±			+
	CD34	−	−	−	+	+	−
Distribution		Bone marrow (Pittenger et al., 1999), adipose tissue (Zuk et al., 2001) and many others tissue cultures (da Silva Meirelles et al., 2006)	Bone marrow cultures (Petrini et al., 2009)	Sub-endothelium of vessels and microvessels in bone marrow (Tormin et al., 2011) and adipose tissue (Corselli et al., 2012)	Adventitia of vessels and microvessels in bone marrow (Sacchetti et al., 2007)	Adventitia of vessel and microvessel in adipose tissue (Tormin et al., 2011; Corselli et al., 2012)	Adjacent to trabecular bone (Tormin et al., 2011)
Role in angiogenesis		Controversial	Sprouting and direct differentiation into ECs	Stabilization of new formed vasculature	Mural cells and endothelium support	Mural cells and endothelium support	Not involved

Therefore, we hypothesize that the presence of the recently described Mesodermal Progenitor Cells (MPCs) could be responsible for the controversial data regarding angiogenic potential of MSC cultures. Although these cells can be co-isolated with MSC culture, different protocols may determine a different yield of MPCs.

The discovery of Nestin-positive CD31-positive MPCs supports their role as MSC ancestors in human bone marrow and indicates the *tunica intima* as a possible source of MSCs.

Further studies are needed to deeply investigate the MPC biology and confirm their anatomical home in human bone marrow. Nonetheless, the identification of MPCs suggests the opportunity of a revision of the MSC definition in order to achieve their expected clinical utility (Keating, 2012).

Finally, MPCs represent a valuable cell population for the proof of new concepts in tissue engineering, where the neo-vascularization plays a crucial role in the establishment of successful therapies. Future studies evaluating MPC-based therapies will take advantage of their mesengenic and angiogenic potential in order to regenerate skeletal tissues and support their growth with a newly formed vasculature.

## Author contributions

Simone Pacini: Literature review and article draft. Iacopo Petrini: revision.

### Conflict of interest statement

The authors declare that the research was conducted in the absence of any commercial or financial relationships that could be construed as a potential conflict of interest.
